# Nineteenth Annual Meeting of the Illinois State Medical Society, Commencing May 18th, 1869, in the City of Chicago

**Published:** 1869-07

**Authors:** 


### NINETEENTH ANNUAL MEETING OF THE ILLINOIS STATE MEDICAL SOCIETY, COMMENCING MAY 18TH, 1869, IN THE CITY OF CHICAGO.

Delegates and members assembled in the Common Council Room, in City Hall, and were called to order at 10 o'clock A.M., by the President, S. T. Trowbridge, of Decatur.

Dr. R. C. Hamill, Chairman of the Committee of Arrangements, welcomed the members in a neat and appropriate address. The Secretary read the names of the delegates and members whose credentials had been presented to the Committee of Arrangements, and who were present, as follows:—

*Delegates.*

*Macon County Medical Society.*—I. N. Barnes, of Decatur.

*McLean County Medical Society.*—S. W. Noble, C. R. Parke, of Bloomington; H. Noble, of Heyworth.

*Adams County Medical Society.*—I. T. Wilson, of Quincy.

*Christian Co. Medical Society.*—B. F. Barnes, of Taylorville.

*Sangamon Co. Medical Society.*—H. B. Buck, of Springfield.

*Quincy Medical Society.*—James H. Reynolds; J. F. McCormick, of Fowler.

*Æsculapian Society.*—G. W. Albin, of Neoga.

*Chicago Medical Society.*—G. C. Paoli, J. M. Hutchinson, A. Fisher, P. S. McDonald, J. S. Hildreth, N. T. Quales, A. Groesbeck, H. P. Merriman, S. J. Avery, Lyman Ware, John G. Fredigke, H. Wanzer, I. N. Danforth.

*McLean County Medical Society.*—John Little, of LeRoy; D. O. Crist, of Bloomington.

*Jersey Co. Medical Society.*—J. O. Hamilton, of Jerseyville.

*Iroquois County Medical Society.*—Elias Wenger, of Gilman.

*Champagne City Medical Society.*—J. T. Pearman, of Champagne.

*DeKalb County Medical Society.*—H. F. Page, of Sycamore.

*Fox River Valley Medical Association.*—C. W. Oleson, of Bloomingdale; A. Hard, of Aurora; R. J. Patterson, of Batavia; D. S. Jenks, of Plano.

*Military Tract Medical Society.*—S. P. Breed, of Princeton.

*Champaign County Medical Society.*—C. H. Mills, of Champaign City.

*Rush Medical College.*—Edwin Powell, DeLaskie Miller, of Chicago.

*Chicago Medical College.*—M. O. Heydock, D. T. Nelson, of Chicago.

*Cook County Hospital.*—J. P. Ross, R. G. Bogue, of Chicago.

*Chicago Eye and Ear Infirmary.*—E. L. Holmes, of Chicago.

*Permanent Members.*

S. T. Trowbridge, of Decatur.

R. C. Hamill, N. S. Davis, E. Ingalls, T. D. Fitch, W. A. Knox, S. A. McWilliams, H. M. Hurd, S. Wickersham, C. T. Fenn, Moses Gunn, J. H. Hollister, H. W. Boyd, R. N. Isham, J. V. Z. Blaney, W. H. Byford, Thos. Bevan, E. O. F. Roler, V. L. Hurlbut, J. Adams Allen, Benj. Durham, of Chicago.

E. Holderness, of Towanda.
D. L. Jewett, of Watseka.
W. R. Fox, of Wilmington.
L. H. Baker, of Quincy.
F. B. Haller, of Vandalia.
E. P. Cook, J. C. Corbus, of Mendota.
A. Niles, of Quincy.
J. P. Matthews, of Carlinville.

On motion, the regular order of business was suspended, for the purpose of electing new members. The following were then proposed in order, and unanimously elected as permanent members:—

W. C. Secrest, of Watseka, Ill.

G. W. Bane, of Macomb, Ill.

D. M. Vosburgh, of Earlville, Ill.

W. M. Burbank, of Barrington, Ill.

E. R. Willard, of Wilmington, Ill.

C. A. W. Zimmerman, of Quincy, Ill.

Peter Epler, of Cayuga, Ill.

John B. Hamilton, of Jerseyville, Ill.

John M. Woodworth, Charles Rutter, S. J. Jones, D. B. Trimble, J. H. Rauch, Wm. C. Lyman, Nelson Loverin, E. Marguerat, Ch. A. Clark, of Chicago, Ill.

R. D. Bradley, of Bloomington, Ill.
Charles Hunt, of Dixon, Ill.
J. R. Corbus, of Amboy, Ill.

On motion of Dr. E. Ingalls, of Chicago, Dr. E. A. Clarke, of St. Louis, Mo., was elected a member, by invitation.

The Secretary then introduced Dr. Hiram Corliss, of Greenwich, Washington Co., New York, as a regularly accredited delegate from the New York State Medical Society. He was welcomed by the President, and invited to take a seat on the platform, to which he responded in an interesting address.

The reports from Standing Committees being in order, were called for as follows:—

*Committee on Surgery*—Dr. A. L. McArthur, of Rockford, Chairman. No report.

The Secretary stated that he received a letter from Mrs. McArthur several weeks since, stating that Dr. McA. had been seriously sick; and although his report was partially prepared, he would not be able to finish it, and requested the next member on the Committee to be informed of the fact. This had been done, but he had heard nothing in reply.

*Committee on Practical Medicine and Epidemics*—Dr. E. P. Cook, of Mendota, Chairman.

Dr. Cook presented his report, which was read at length. Dr. Corliss, of New York, made some remarks in relation to phthisis pulmonalis, expressing his belief in its curability, and encouraging further investigations concerning it.

Dr. E. Ingalls, of Chicago, called attention to the subject of sun-stroke; claiming that its true pathology was *exhaustion*, requiring stimulants, and warm applications to the head.

On motion, the report of Dr. Cook was accepted, and referred to the Committee of Publication.

Committee on Obstetrics—Dr. H. B. Buck, of Springfield, Chairman. The report was made the special order for 2½ o'clock P.M.

Committee on Drugs and Medicines—Dr. N. S. Davis, Chairman. Report, special order for 12 M., of Wednesday.

Dr. J. H. Hollister, Treasurer of the Society, presented the following Annual Report, which was accepted and referred to the Auditing Committee.

The Auditing Committee subsequently reported the accounts and vouchers of the Treasurer correct:—

*Treasurer's Report.*

The Treasurer of the Illinois State Medical Society herewith submits his Seventh Annual Report, in form as follows, for the year ending May, 1869:—

JOHN H. HOLLISTER, *Treasurer*,
In acc't with ILL. STATE MED. SOCIETY.

| | | | |
|---|---|---|---|
| *1868.* | *Dr.* | | |
| *May* | Balance of cash in Treasury at date,----- | | $ 43 39 |
| | Amount of cash received at the Annual Meeting at Quincy, in May, 1868. | | |
| *1869.* | T. D. Fitch, Treas., P. T.,------------ | | 166 00 |
| *May 18.* | Am't received by Treasurer in answer to correspondence with absent members to date,------------------------------- | | 45 00 |
| | Making the am't in full,---------- | | $254 39 |
| | *Cr.* | | |
| | By cash paid for printing the Annual Transactions for 1868, as per voucher, -------- | $169 68 | |
| | Cost of correspondence by Treasurer,------ | 3 00 | 172 68 |
| | Leaving a balance in the Treasury of---- | | $81 71 |

It should be stated, that so far as relates to the expense of mailing the Transactions to the members who had paid their dues in advance, it has been made a matter of gratuity, by the generosity of your Permanent-Secretary. The expenditure for the printing of Transactions for 1868 being less than for those of former years, your Treasurer is enabled to report a favorable balance; and, yet, it is believed that an average assessment of $3 per annum, and a very general payment of annual dues, will be needed to maintain the financial credit of the Society.

Respectfully submitted,

J. H. HOLLISTER, *Treasurer.*

*The Secretary*, in behalf of the Committee of Publication, made the following report, which was accepted and placed on file:—

The undersigned, on behalf of the Committee of Publication, would respectfully report as follows:—As soon after the papers and reports presented at the last meeting of the Society were in possession of the Committee, as possible, they were put to press. Those presented by Dr. E. Powell were not placed in the hands of the Committee, and, consequently, could not appear in the Transactions. Two hundred copies were printed, and copies sent to all who were reported as having paid the annual assessment, and also to the several medical journals in the country. Near one hundred copies are left, thirty of which are in the hands of the Treasurer, and the balance are in possession of the Permanent-Secretary. The cost of the publication was $169 68, as will appear from the report of the Treasurer.

N. S. Davis, *Permanent-Secretary.*

The list of Special Committees was called, but only the following were responded to:—

Committee on Ophthalmology—Dr. E. L. Holmes, Chairman. Report ready, and made special order for to-morrow, at 11 o'clock A.M.

Committee on Cholera Infantum—Dr. N. Wright, of Chatham, Chairman, reported progress, and asked to be continued. On motion, the Committee was continued to report at next annual meeting.

Committee on Staphyloraphy—Dr. Moses Gunn, of Chicago, Chairman. Report ready, and made the special order for 3 o'clock P.M.

Dr. D. L. Jewett, of Watseka, member of the Committee on Practical Medicine, read a paper as a supplement to the report of the Chairman of that Committee. On motion of Dr. S. W. Noble, the paper was accepted and referred to the Committee of Publication. The Chairman of the Committee of Arrangements read invitations from the Cook County Hospital, the Mercy Hospital, the Marine Hospital, the Chicago Medical College, and the Rush Medical College, to visit those institutions, at such times as the Society might deem proper.

On motion of Dr. F. B. Haller, the invitations were accepted, and the time and order of visiting the several institutions referred to the Committee of Arrangements.

An invitation was extended to all the members of the Society, and their ladies, to attend a reception at the residence of Dr. N. S. Davis, on Wednesday evening.

On motion of Dr. S. W. Noble, the delegates from each county were instructed to select one of their number to act as a member of the Committee on Nominations, and to report the same to the Secretary on the reassembling of the Society at 3 o'clock P.M. Adjourned.

AFTERNOON SESSION.

3 o'clock P.M., Society called to order, the President, Dr. S. T. Trowbridge in the Chair.

The Secretary stated that the following names had been reported as members of the Nominating Committee:—

G. W. Abbin, Cumberland County; A. Niles, Adams Co.; R. G. Bogue, Cook Co.; H. Noble, McLean Co.; W. R. Knox, Will Co.; F. B. Haller, Fayette Co.; J. T. Pearman, Champaign; J. C. Corbus, LaSalle; B. F. Barnes, Christian; J. P. Matthews, Macoupin Co.; G. H. Bane, McDonough Co.; H. F. Page, DeKalb Co.; H. B. Buck, Sangamon Co.; J. N. Barnes, Macon Co.; Elias Wenger, Iroquois Co.; J. O. Hamilton, Jersey Co.; P. Epler, Livingston Co.

Dr. H. B. Buck, of Springfield, Chairman of the Committee on Obstetrics and Diseases of Females, read his report at length.

Dr. Corliss, of New York, remarked that his experience had led him to regard placenta prævia as of very rare occurrence. He also related two cases of inversion of the uterus. The first died from loss of blood, within five minutes after his arrival. In the second case, the womb was returned to its natural position, and the patient recovered. The report of Dr. Buck was accepted and referred to the Committee of Publication.

Dr. N. S. Davis having filled the office of Permanent-Secretary *twelve* years tendered his resignation.

On motion of Dr. H. Noble, the resignation was accepted, and the thanks of the Society unanimously tendered to Dr. Davis, for the faithful manner in which he had so long discharged the duties of his office.

Dr. Hamill, Chairman of the Committee of Arrangements, announced that Dr. E. A. Clarke, of St. Louis, Mo., would exhibit to the Society, at 9 o'clock A.M., models of his improved apparatus for dressing fractures of the extremities, etc.

Dr. Hurd, of Galesburg, stated that he had nearly completed his special report on Aphasia, when his work was interrupted by sickness.

On motion of Dr. T. D. Fitch, Dr. Hurd was requested to complete his report and transmit it to the Committee of Publication.

Dr. Moses Gunn read his report on Staphyloraphy, in which the surgical operations hitherto performed for the closure of cleft-palate were described, and the benefits resulting therefrom impartially considered. The chief benefit to be derived from an operation in these cases is the improvement of speech. And in this respect the writer thought the amount of benefit resulting in the majority of cases was very small; so small, indeed, as to render the operation one of doubtful propriety.

Dr. Corliss mentioned a case operated on by Dr. G. Buck, of New York, and presented before the New York State Medical Society, in which the speech was restored very perfectly, with the aid of an instrument fitted by Dr. Kingsbury, a dentist of that city.

Dr. Fitch stated that a paper had been read on this subject at the recent meeting of the American Medical Association, by Dr. Whitehead, of New York; and the conclusions arrived at in reference to the benefits resulting from surgical operations were similar to those in the report just read.

Dr. Davis suggested that the improvement in speech made in the case presented by Dr. Buck was owing to the dental instrument, and not to the surgical operation performed by Dr. Buck.

On motion, the report of Dr. Gunn was accepted and referred to the Committee of Publication.

Dr. J. S. Hildreth, of Chicago, read a paper on the means for improving the results of the operations for cataract. After some remarks from Dr. E. Powell, the paper of Dr. Hildreth was referred to the Committee of Publication.

A resolution from the McLean County Medical Society was read, inviting the State Society to hold its next annual meeting at Bloomington. It was referred to Committee on Nominations.

Dr. J. O. Hamilton, of Jerseyville, exhibited a specimen of calcarious matter, from the uterine surface of a placenta. Dr. G. W. Albin, of Neoga, presented a malformed female fœtus, with a slender projection from the forehead, somewhat resembling a penis.

On motion, the Society adjourned until 9 o'clock A.M.

WEDNESDAY, JUNE 19TH, 1869—SECOND DAY.

The Society was called to order by the President, at 9 o'clock A.M. The reading of the minutes was dispensed with, and the special order for the morning taken up.

Dr. E. A. Clarke, of St. Louis, presented his apparatus for the treatment of fractures of the upper and lower extremities and of the lower jaw, and gave an interesting account of their application. Dr. Corliss made some remarks in regard to the advantage of having surgical appliances simple as possible in their construction, and strongly commended the use of starch bandages in the treatment of fractures.

On motion of Dr. Hamill, a vote of thanks was given to Dr. E. A. Clarke, for the exhibition of his apparatus.

Dr. A. Van Dyke, of Oswego, New York, was introduced to the Society by Dr. Corliss, and was elected a member by invitation.

The Committee on Nominations, through the Secretary, made a report.

Dr. J. C. Corbus, of Mendota, moved that the report of the Committee be amended, by striking out Bloomington and inserting Dixon, as the next place of meeting. After some discussion, in which Drs. Corbus, Hunt, of Dixon, Cook, H. Noble took part, the amendment was carried by a vote of ayes 16, noes 15. The report of the Committee was then referred back for attention of the Committee of Arrangements and Assistant-Secretary. It was again reported and adopted as follows:—

Chicago, Ill., 18th May, 1869.

The Committee on Nominations met and organized by electing Dr. H. Noble, Chairman, and Dr. J. O. Hamilton, Secretary.

Proceeded to put in nomination for the ensuing year the following named officers:—

*President*—Prof. J. V. Z. BLANEY, of Chicago.

*1st Vice-President*—Dr. G. W. ALBIN, of Neoga.

*2d Vice-President*—Dr. H. B. BUCK, of Springfield.

*For Treasurer*—Dr. J. H. HOLLISTER, of Chicago.

*Permanent-Secretary*—Dr. T. D. FITCH, of Chicago.

*Assistant-Secretary*—Dr. Charles HUNT, of Dixon.

*Place of Meeting*—Dixon.

The following Committees were nominated:—

*On the Practice of Medicine*—Drs. C. Goodbrake, of Clinton; James S. Whitmire, of Matamora; and Ira Barnes, of Decatur.

*On Surgery*—Drs. Moses Gunn, of Chicago; G. R. Bibb, of Jacksonville; and John B. Hamilton, of Jerseyville.

*On Obstetrics*—Drs. Elias Wenger, of Gillman; J. T. Pearman, of Champaign; and S. P. Breed, of Princeton.

*On Drugs and Medicines*—Drs. Charles Hunt, of Dixon; C. R. Parke, of Bloomington; and T. G. Hickman, of Vandalia.

*On Necrology*—Drs. J. H. Hollister, of Chicago; F. B. Haller, of Vandalia; and J. O. Hamilton, of Jerseyville.

Special Committees:—

*On Criminal Abortions*—Drs. DeLaskie Miller, of Chicago; W. H. Byford, of Chicago; and H. Noble, of Bloomington.

*On Ophthalmology*—Drs. J. S. Hildreth, of Chicago; E. L. Holmes, of Chicago; and S. J. Jones, of Chicago.

*On Pulmonary Tuberculosis*—Drs. J. P. Ross, of Chicago; J. N. Niglas, of Peoria; and T. D. Washburn, of Hillsboro.

*On the use of Plaster-Paris in Fractures*—Dr. R. G. Bogue, of Chicago.

*On Otology*—Drs. Samuel J. Jones, of Chicago; J. R. Corbus, of Amboy; and J. S. Hildreth, of Chicago.

*On Arrangements*—Drs. Everett, Phillips, Hunt, of Dixon; J. C. Corbus, of Amboy; and E. P. Cook, of Mendota.

H. NOBLE, *Chairman.*

J. O. HAMILTON, *Secretary.*

On motion, a vote of thanks was tendered to the McLean Co.

Medical Society for its invitation to hold the next Annual Meeting in Bloomington.

Dr. Edwin Powell, of Chicago, presented a special report on Lithotomy, giving only a verbal synopsis. After some remarks by Dr. E. A. Clarke, and others, the report was referred to the Committee of publication.

Dr. N. S. Davis, of Chicago, read the report from the Standing Committee on Drugs and Medicines.

Dr. J. V. Z. Blaney remarked that the Peroxide of Hydrogen acted on the animal organization by super-oxydation of all the tissues. He explained the distinction between the Ozonized or active Oxygen, and the Beta or passive Oxygen. He further suggested that the Permanganate of Potassa in solution possessed all the medicinal properties of the Peroxide Hydrogen, and was accessible to the whole profession.

Dr. Davis suggested that the Potassa in the Permanganate might be objectionable in some aplastic conditions of the system. Dr. Blaney replied that the addition of a few drops of Nitric Acid to the solution would obviate any objection of that kind.

Dr. E. Ingalls, of Chicago, stated that he had used the Permanganate of Potassa with great benefit in bad cases of Diphtheritic deposits in the throat and air-passages.

Dr. M. O. Heydock, of Chicago, stated that of all the cases of Diabetes treated with Permanganate of Potassa and Peroxide Hydrogen, only one to two appear to have been really successful. He had used the Permanganate of Potassa in Diphtheria with only indifferent success. Had used a solution of from three to four grains to the ounce of water, as a douche in nasal catarrh with offensive discharge, with much benefit.

On motion the report was referred to the Committee of Publication.

Dr. E. L. Holmes, Chairman of the Committee on Ophthalmology, presented and read his report, which was refered to the Committee of Publication.

On motion the Society adjourned until nine o'clock, A.M., the afternoon to be spent in visiting the Hospitals and Colleges.

At half-past two o'clock P.M., the Members of the Society, under the direction of Drs. Hamill and Davis, took the street cars for the County Hospital. They were received and conducted through that institution by Drs. Fitch, Bevan, and Hildreth, of the Hospital Staff. They next made a short visit to the Chicago Medical College, where they were received and conducted through the Institution by N. S. Davis, President of the College. At four o'clock they arrived at the Hospital of the Sisters of Mercy, where they were received by Drs. Andrews and Davis, and after examining the different wards they were conducted into the reception room, where the sisters had unexpectedly provided a generous collation. After having done ample justice to this, and given a vote of thanks in return, they again entered the street cars and proceeded to the Marine Hospital, where they were cordially received by Dr. E. C. Rogers and his Assistants. From thence they proceeded to the Rush Medical College, and were kindly shown through its halls by Dr. E. Ingalls. This closed the work of the afternoon.

In the evening, the Members of the Society and a few invited guests, enjoyed a very pleasant social entertainment at the residence of Dr. N. S. Davis.

THURSDAY MORNING—THIRD DAY.

The Society was called to order at nine o'clock A.M., by the President. The reading of the minutes by the Secretary was dispensed with.

Dr. E. Ingalls exhibited an instrument for returning the *funis* when prolapsed and retaining it until the labor is sufficiently advanced. He also read a report on *Lenient Medication*, or Conservatism in Medicine.

The report was received and referred to the Committee of Publication.

Dr. J. H. Hollister, Chairman of the Committee on Necrology, read a report embracing a biographical sketch of the late Prof. Wm. B. Herrick, one of the founders of this Society. The report was accepted and referred to the Committee of Publication.

Dr. E. Andrews, of Chicago, read a short report on the value of mixing Oxygen with Nitrous Oxide for inhalation as an anæsthetic. He gave the results of several experiments tending to establish the fact that the addition of a small proportion of Oxygen to Nitrous Oxide rendered it less dangerous for protracted use, and yet did not prevent it from producing complete anæsthesia.

Dr. T. D. Fitch discussed the comparative danger from the use of Chloroform and Ether; expressing the opinion that the latter was very little less dangerous than the former.

Dr. Andrews thought the danger from inhalation of Ether only one tenth that from Chloroform.

Dr. E. Powell stated the fact that a few years since he saw the Ether administered to a patient in the Hospital at Boston, Massachusetts, who died directly from the effects of the inhalation.

Dr. B. Durham mentioned the case of a lady who inhaled Ether several months since, and is still suffering severe cerebral symptoms in consequence.

Dr. J. S. Hildreth stated that the inhalation of absolutely pure Ether was quite safe, but many of the samples found in the drug stores were unfit to use.

Dr. Moses Gunn thought the gases had not yet been tested sufficiently, and that we must, at present, choose between the Chloroform and Ether as anæsthetics for surgical purposes. Until within the last six months he had used Chloroform almost exclusively, but had been finally induced to give Ether the preference on account of its greater safety and more stimulating qualities.

Dr. J. H. Hollister thought a mixture of two parts Ether and one Chloroform preferable to either alone.

Dr. J. S. Jones coincided with Dr. Hollister.

Dr. J. V. Z. Blaney stated that he had examined many specimens of Ether from the best drug houses here, and such as is being used for inhalation. The best specimens contained only from 60 to 70 per cent of pure Ether. The specimens of Chloroform were more nearly pure.

Dr. E. Ingalls thought the paper of Dr. Andrews calculated to produce unnecessary fears by placing the ratio of deaths from Chloroform too high.

Dr. Bane related a case of death from Chloroform that he saw under the care of Dr. F. H. Hamilton, of New York.

The paper of Dr. E. Andrews was referred to the Committee of Publication.

The report of Dr. R. J. Patterson, Chairman of the Committee on Insanity, was received and read by the Secretary, and referred to the Committee of Publication.

Dr. E. Ingalls offered the following resolution, which was unanimously adopted—

*Resolved*, That in the opinion of the Members of this Society the Legislation recommended by the Committee on Insanity is judicious.

On motion of Dr. Blaney, a Committee of three was appointed to memorialize the next Legislature in favor of the passage of the law recommended in the report and endorsed by the foregoing resolution.

The President appointed as such Committee Drs. R. J. Patterson, of Batavia, E. Ingalls, of Chicago, and H. H. Roman, of Springfield,

Dr. Corliss, the delegate from the New York State Medical Society, in taking leave of the Society made some appropriate remarks.

Dr. E. Ingalls responded, and offered a vote of thanks to the New York State Society for sending a delegate to meet with us, which was unanimously adopted.

On motion, Dr. W. C. Lyman, of Chicago, was appointed a Special Committee on Laryngoscopy.

Dr. T. D. Fitch called up the amendments to the constitution of the Society proposed by him at the last annual meeting, and printed in the transactions for 1868.

On motion, the first section as printed was unanimously adopted as an additional clause to the present constitution.

The second section being under consideration, Dr. E. Ingalls moved to strike out all relating to Membership of Local Societies. The motion led to a discussion, which was participated in by Drs. Ingalls, Hollister, Fenn, Fitch, Davis, Blaney, Haller, Hildreth, and Miller.

Dr. Hildreth called for a division of the question, but on motion the second and third clauses of the proposed amendments were laid on the table until the next Annual Meeting of the Society.

Dr. N. S. Davis, from the Special Committee appointed for that purpose, laid before the Society the following communication from the American Medical Association :—

"Dr. Davis, Chairman of the Committee on Various Propositions and Communications from Medical Societies, etc., offered the following report. The resolutions were adopted and referred to the Committee of Publication:—

*Report of the Committee on Various Propositions and Communications from Medical Societies, etc.*

After a fair examination of the various propositions concerning medical education, medical legislation, and ethical regulations for medical colleges, referred to your committee, we respectfully submit the following report:—

Whereas, The history of medical legislation in the various States of this Union, clearly shows that no reliance can be placed on either the uniformity or the permanency of any laws relating to the practice of medicine; and

Whereas, The results of all the efforts made during the last twenty-five years, to elevate the standard of medical education through concert of action among the numerous medical colleges of this country, have proved with equal clearness that such concert of action in an efficient manner is unattainable, therefore be it

*Resolved*, That whatever is done to establish and maintain a just and fair standard of medical education throughout our whole country, must be done by the profession itself, through its own voluntary organizations, in the same manner that it now establishes and enforces its code of ethics. The profession is as competent to declare, through its representatives in the national, state, and local societies, what shall be the standard of attainments for those to be recognized and admitted into its ranks, and to establish the boards or agencies by which compliance with such standard shall be ascertained, as it is to declare what shall be the ethical rules governing the conduct of those already admitted.

*Resolved*, That this Association earnestly requests each State Medical Society to appoint annually one or more Boards of Examiners, composed of five thoroughly competent members, whose duty it shall be to meet at suitable times and places, for the examination of all persons, whether graduates of colleges or not, who propose to enter upon the practice of medicine in their respective States, except such as have been previously examined and licensed by a similar Board in some other State.

*Resolved*, That each State Medical Society be requested to make such regulations concerning the pay of the Boards of Examiners, and the fee to be charged for a license to practise, that the former shall in no case depend on the amount received from the latter.

*Resolved*, That each State Medical Society be requested to require its Examining Board or Boards to exact of every applicant for examination, adequate proof that he has a proper general education; is twenty-one years of age, and has pursued the study of medicine three full years, one-half of which time shall have been in some regularly organized Medical College, whose curriculum embraces adequate facilities for didactic, demonstrative, and hospital clinical instruction.

*Resolved*, That each State Medical Society be requested to act on the foregoing propositions at the next regular annual meeting after the reception of copies of the same, and if approved and adopted by the State Medical Societies of two-thirds of the States, this Association shall deny representatives from all organizations who longer refuse to comply with the same, and shall recommend the State Societies to do the same, and all persons who, after that date, seek to enter upon the practise of medicine without first receiving a license from State Board of Examiners, shall be treated ethically as irregular practitioners.

*Resolved*, That in adopting the foregoing resolutions, by which it is proposed to treat the medical college diploma the same as the diploma of any literary college, this Association is actuated by no desire to injure the medical schools of our country. On the contrary, by the adoption of the fourth resolution at the same time that the value of the mere college diploma is practically nullified, it is the desire, and confident expectation, that those institutions will be greatly benefited; because they will be forced to rival each other in the extent and efficiency of their courses of instruction, instead of the number of diplomas which they can annually distribute.

All of which is respectfully submitted.

N. S. DAVIS,
PAUL F. EVE,
E. S. GAILLARD,
E. LEE JONES,
J. K. BARTLETT.

On motion of Dr. Davis, it was

*Resolved*, That a special committee of three be appointed by the President to present copies of the resolutions adopted before the several State Medical Societies at as early a period as possible.

*Committee*—Dr. Davis, Illinois; J. S. Weatherly, Alabama; J. M. Toner, District of Columbia."

After the communication had been read, owing to the importance of the subject and the nearness of the hour of final adjournment, it was moved that the preamble and resolutions be referred to a Special Committee of three, with instructions to report at the next Annual Meeting of this Society. The motion was adopted, and the President appointed as such Committee Drs. N. S. Davis, of Chicago, David Prince, of Jacksonville, and S. W. Noble, of Bloomington.

Dr. J. S. Hildreth offered the following resolution as an addition to the bye-laws of the Society:—

*Resolved*, That it shall be the duty of the Chairman of every Committee of this Society to invite all other Members of his Committee, at least three months before the next Annual Meeting of the Society, to contribute to the report to be made by the Committee. And further, he shall submit the report to all other Members of the Committee who have signified a desire to take part in the same, for their action.

The resolution was adopted.

The Society then proceeded to elect delegates to various Medical Societies.

Delegates to the American Medical Association—N. S. Davis, Chicago; F. B. Haller, Vandalia; DeLaskie Miller, Chicago; H. Noble, Heyworth; Benj. Durham, Chicago; Dr. Vosbury, Earlville; James H. Reynolds, Payson, Adans Co.; S. P. Breed, Princeton; D. L. Jewett, Watseka; R. J. Patterson, Batavia; J. O. Hamilton, Jerseyville; D. S. Jenks, Plano, Kendal Co.; M. C. Lyman, Chicago; J. V. Z. Blaney, Chicago; Samuel J. Jones, Chicago; T. D. Fitch, Chicago; A. H. Luce, Bloomington; H. B. Buck, Springfield; Oliver Everett, Dixon; G. W. Albin, Neoga.

Delegates to New York State Medical Society—R. C. Hamill, Chicago; Dr. Thomas, Chicago.

California State Medical Society—Drs. E. Ingalls, Chicago; S. J. Jones, Chicago.

Missouri State Medical Society—J. O. Hamilton, Jerseyville; F. B. Haller, Vandalia.

Michigan State Medical Society—Moses Gunn, Chicago; E. Andrews, Chicago.

Wisconsin State Medical Society—J. H. Rauch, Chicago; Wm. C. Lyman, Chicago.

Indiana State Medical Society—E. P. Cook, Mendota, Ill.; Wm. M. Chambers, Charleston.

Iowa State Medical Society—H. M. Hurd, Galesburg, Ill.

Dr. N. S. Davis offered the following resolutions, which were unanimously adopted:—

*Resolved*, That the thanks of the State Medical Society are hereby tendered to the Mayor and Common Council of the city of Chicago for the free use of the Council Chamber for the present Annual Meetings of the Society.

*Resolved*, That the thanks of this Society are cordially tendered to the Committee of Arrangements for the judicious provisions they have made for the accommodation of the Society.

*Resolved*, That the thanks of this Society are hereby tendered to our retiring President for the able and efficient manner in which he has performed his official duties.

Dr. J. H. Hollister moved a vote of thanks to the several Colleges, Hospitals, and other Institutions from which this Society had received invitations and professional courtesies, which motion was adopted.

On motion the thanks of the Society were tendered to Dr. N. S. Davis and Lady for the very pleasant entertainment enjoyed at their residence on Wednesday evening.

The following were appointed a Board of Censors for the ensuing year:—Drs. E. P. Cook, of Mendota; J. O. Hamilton, of Jerseyville; S. P. Breed, of Princeton; N. Wright, of Chatham; and E. Powell, of Chicago.

On motion the Society adjourned *sine die*.

